# In striatum phosphodiesterase 10A is part of a synaptic signalling complex

**DOI:** 10.1186/2050-6511-14-S1-P59

**Published:** 2013-08-29

**Authors:** Corina Russwurm, Doris Koesling, Michael Russwurm

**Affiliations:** 1 Institut für Pharmakologie und Toxikologie, Medizinische Fakultät, MA N1, Ruhr-Universität Bochum, Bochum, Germany

## Background

A number of neurological disorders (i.e. Schizophrenia and Parkinson's disease) that result from dysfunction of striatal signal transduction pathways underline the importance of the striatum for motor function and procedural learning. Most striatal neurons are medium spiny neurons (MSN) that receive input via dopaminergic and glutamatergic terminals and project to the basal ganglia. The MSN express dopamine receptors that are either positively (D1) or negatively (D2) coupled to adenylyl cyclase therefore dopamine directly influences intracellular levels of cAMP. Participants in cAMP mediated signalling pathways are often organized in multiprotein complexes around A-kinase anchoring proteins (AKAPs). The proximity of the cyclases and phosphodiesterases (PDEs) as well as protein kinase A ensures fine tuning of cAMP levels in a given compartment and allows for selective regulation of target proteins such as NMDA or AMPA receptors by phosphorylation.

## Results and conclusion

According to our results PDE10 is highly expressed in striatum and responsible for 70% of cAMP degrading activity. Therefore we asked if PDE10 participates in signalling complexes at the synapse. Indeed, the immunoprecipitation via PDE10 specific antibodies revealed interactions with AKAP150 and AKAP-interacting proteins as well as subunits of the NMDA-receptor and PSD95. In the isolated complex and in striatal slices, PDE10 can be phosphorylated by activation of PKA. This phosphorylation is short lived, probably because it is reversed by permanently active protein phosphatase 1. We observed that dephosphorylation is additionally accelerated via NMDA dependent rise in intracellular Ca^2+^ concentrations.

